# Transcriptome Co-expression Network and Metabolome Analysis Identifies Key Genes and Regulators of Proanthocyanidins Biosynthesis in Brown Cotton

**DOI:** 10.3389/fpls.2021.822198

**Published:** 2022-02-14

**Authors:** Zhenzhen Wang, Xiaomeng Zhang, Shoupu He, Abdul Rehman, Yinhua Jia, Hongge Li, Zhaoe Pan, Xiaoli Geng, Qiong Gao, Liru Wang, Zhen Peng, Xiongming Du

**Affiliations:** ^1^State Key Laboratory of Cotton Biology, Institute of Cotton Research, Chinese Academy of Agricultural Sciences, Anyang, China; ^2^Zhengzhou Research Base, State Key Laboratory of Cotton Biology, Zhengzhou University, Zhengzhou, China; ^3^National Nanfan Research Institute (Sanya), Chinese Academy of Agricultural Sciences, Sanya, China

**Keywords:** brown cotton, transcriptome, metabolome, flavonoid metabolism, yeast one-hybrid, R2R3-MYB genes

## Abstract

Brown cotton fiber (BCF) is a unique raw material of naturally colored cotton (NCC). But characteristics of the regulatory gene network and metabolic components related to the proanthocyanidins biosynthesis pathway at various stages of its fiber development remain unclear. Here, the dynamic changes in proanthocyanidins biosynthesis components and transcripts in the BCF variety “Zong 1-61” and its white near-isogenic lines (NILs) “RT” were characterized at five fiber developmental stages (0, 5, 10, 15, and 20 days post-anthesis; DPA). Enrichment analysis of differentially expressed genes (DEGs), comparison of metabolome differences, and pathway enrichment analysis of a weighted gene correlation network analysis together revealed the dominant gene expression of flavonoid biosynthesis (FB), phenylpropanoid metabolisms, and some carbohydrate metabolisms at 15 or 20 DPA than white cotton. Eventually, 63 genes were identified from five modules putatively related to FB. Three R2R3-MYB and two bHLH transcription factors were predicted as the core genes. Further, *GhANS*, *GhANR1*, and *GhUFGT2* were preliminarily regulated by *GhMYB46*, *GhMYB6*, and *GhMYB3*, respectively, according to yeast one-hybrid assays *in vitro*. Our findings provide an important transcriptional regulatory network of proanthocyanidins biosynthesis pathway and dynamic flavonoid metabolism profiles.

## Introduction

Naturally colored cotton (NCC) is a variety of cotton with natural pigmentation in its fiber. The most common fiber colors are brown and green (Dutt et al., 2004). Compared with traditional cotton plants, colored ones provide significant benefits, namely a higher amount of tannin and phenols, and also resistance against diseases and insects. Because NCC fiber has a natural color, it does not undergo chemical bleaching and dyeing during textile processing. This advantage not only reduces environmental pollution and the harm to human health, but also decreases cost and increases the revenue of cotton growers. Indeed, NCC is considered as ecologically and environment-friendly cotton products with broad prospects (Hua et al., 2007; Efe et al., 2009; Khatri et al., 2015). However, the quality of its colored cotton is not as good as that of white cotton. Likewise, single color and unstable pigment heredity have restricted its production, promotion, and end-use (Feng et al., 2011, 2015). In recent years, breeders have developed some commercial color cotton genotypes with good fiber quality. Yet, due to the lack of NCC genetic resources, traditional breeding programs are reluctant to change the color type (Sun et al., 2021). Therefore, genetic engineering or gene-editing technology is considered a promising tool to produce new types of fiber color.

Brown cotton is considered the most common type of NCC. The early identification of its chemical components showed that flavonoids were responsible for this brown color of cotton fiber (Xiao et al., 2007; Hua et al., 2009). Moreover, procyanidins (PAs) have proven to be the main component of pigment component in brown cotton fibers (BCFs; Li et al., 2013; Feng et al., 2014; Xiao et al., 2014). Additionally, *leucoanthocyanidin reductase* (*LAR*) plays a crucial role in the PA biosynthesis pathway of brown fibers, and its metabolites catechin and epicatechin are the main precursors of condensed tannins. *Anthocyanidin reductase* (*ANR*) is a key structural gene in the PA pathway (Feng et al., 2014). Recently, these two genes, *LAR* and *ANR*, have been verified for their transgene functionality, enabling that the development of new colored cotton germplasm has been developed (Gao et al., 2019). In the last decade, transcriptomic or proteomic analyses were used to uncover the PA precursor synthesis pathway involved in fiber pigmentation. It entails a group of flavonoid pathway genes, including those for the particular enzymes *LAR* and *ANR* of two PA precursors, all of which have been found upregulated or deposited in brown cotton (Xiao et al., 2007, 2014; Feng et al., 2013, 2014; Li et al., 2013; Gong et al., 2014; Hinchliffe et al., 2016; Peng et al., 2020).

The color of BCF is a genetically determined feature, which is produced by condensed tannins accumulating in the lumen of cotton fiber (Carvalho et al., 2014; Feng et al., 2014; Gong et al., 2014). Early work by Kohel (1985) tested the allele of the known lint color gene, which finds that the brown fiber color may be controlled by six loci, namely, *Lc1* to *Lc6*. Whereas *Lc1* and *Lc2* are responsible for lint that is medium-brown, *Lc3* is responsible for its dark brown appearance, and *Lc4*, *Lc5*, and *Lc6* are responsible for light-brown coloring (Wang et al., 2014). Simple sequence repeat (SSR) markers revealed that *Lc1* is located on chromosome A07 in upland cotton, whereas *Lc2* was detected on chromosome A06 (Wang et al., 2014). Later, Hinchliffe et al. (2016) found that *Lc1* is *GhTT2_A07*, which is a transcription factor (TF) similar to *Arabidopsis TRANSPARENT TESTA 2* (*TT2*). More recently, Wen et al. (2018) and Yan et al. (2018) elucidated the critical regulatory mechanisms of the *GhTT2* gene involved in the color of BCF *via* its fine mapping, gene expression difference analysis, and transgenic function verification. To date, only *Lc1* gene is well understood, whereas the mechanisms of the other five Lc genes are still unknown.

Few studies have investigated the relationship between proanthocyanidins biosynthesis and metabolism in cotton fiber and their effects on metabolism-related functional genes. In previous research, the metabolic pathways and key proteins involved in BCF’s development and pigment biosynthesis were identified through proteomic and metabolomic analyses, for which selection criteria consisted of the fiber elongation and secondary wall thickening phases (Peng et al., 2020). In this study, we instead focused on early fiber development (0–20 DPA, different nodes of fiber initiation and elongation), using transcriptome and non-targeted metabolome analyses to identify key genes, metabolites, and coexpression networks involved in proanthocyanidin biosynthesis, and obtained pivotal TF genes *via* yeast one hybrid. The key TFs or genes active in the gene coexpression network of transcriptional regulation as related to brown color of cotton fiber, and likewise for other metabolic pathways related to fiber quality formation, are then discussed. The understanding of molecular mechanisms of pigmentation in brown cotton is still limited. Therefore, characterizing the metabolic pathways or transcriptome as related to PA biosynthesis in BCF by high-throughput sequencing technology is instrumental for mining prominent genes for the use in brown cotton breeding, and also for studying the corresponding relevant transcriptional regulation mechanisms.

## Materials and Methods

### Plant Materials and Tissue Collection

Two upland cotton genotypes were used, Zong 1-61 (Abb. Z161), natural brown fiber cotton, and RT-white fiber (Abb. RT), whose cotton fibers are white. RT is a near-isogenic line (NIL) of Z161, as shown by Peng et al. (2020). The eight agronomic traits and four fiber color characters of Z161 and RT, namely, boll weight (BW), micronaire (MIC), fiber elongation (FE), seed-index (SI), lint percentage (LP), fiber length (mm) (FL), fiber strength (cN/tex) (FS), fiber length uniformity (LU), total chromatic aberration (ΔE), chromaticity difference (ΔC), chromaticity index a (Δa), and chromaticity index b (Δb) were determined from six environments, including Anyang of Henan Province; Alaer and Kuitun of Xinjiang Province in 2018 and 2019 with three replications. Young cotton bolls were labeled using tags on their day of anthesis, with their cotton ovule, and fibers were harvested at 0, 5, 10, 15, and 20 DPA for total RNA extractions. The harvested fibers (or ovules) were stripped and immediately frozen in liquid nitrogen and stored at −80°C for the RNA extraction.

### Detection of Color Difference in Cotton Fiber

Fiber samples of the two accessions were collected from three experimental sites (cities of Anyang, Alaer, and Kuitun in China) at maturity in 2019. The whiteness colorimeter YT-48A (Yante Technology Co., Ltd., Hangzhou, China) was used to determine the color difference value of a given fiber. That instrument can measure the color and color difference reflected by the object (paper, fiber, etc.), the CIE brightness (i.e., Ganz brightness W10 and color deviation TW10), the white of Hunter System Lab and Hunter (Lab), yellow, opacity, transparency, light scattering coefficient, and light absorption coefficient of a given fiber sample. In this study, the whiteness of cotton fiber hunt (L, a, b) was measured (Ibraheem et al., 2012; Karaarslan et al., 2013). The total chromatic aberration (ΔE), chromaticity difference (ΔC), chromaticity index a (Δa), and chromaticity index b (Δb) were used to evaluate the cotton fiber color difference.

### RNA Extraction, cDNA Library Construction, and Sequencing

Total RNA of the two cotton genotypes from the 0, 5, 10, 15, and 20 DPA fiber samples were isolated with the RNAprep Pure Plant Plus Kit (DP441) (Tiangen, Beijing, China) following the manufacturer’s protocol. The quality and concentration of each RNA sample were checked using an Agilent 2100 Bioanalyzer (Agilent Technologies, Palo Alto, CA, United States) with 28S/18S ≥ 1.5, A260/A280 ≥ 2.0, and RIN ≥ 7.5. High-quality RNA was prepared to construct a cDNA library, each with three biological replicates.

The 30 cDNA libraries’ construction and sequencing were performed by the BGI Gene Technology Co. (Shenzhen, China). Finally, the cDNA libraries were sequenced on the BGISEQ-500 sequencing platform, and 150-bp paired-end (PE150) reads were generated.

### Mapping Onto the Cotton Genome and Quantification of Gene Expression

The sequences of the adaptor, any unknown bases, and all low-quality reads were removed using the SOAP nuke (v1.4.0, parameters --l 5, -q 0.5, -n 0.1)^1^. Then, these clean reads were saved in a FASTQ format. Next, the clean reads of all the 30 samples were mapped onto the *Gossypium hirsutum* (TM-1) reference genome files downloaded from https://www.cottongen.org/data/download/genome_tetraploid/AD1 (CRI v1) by Bowtie2 (Langmead and Salzberg, 2012) and HISAT (Kim et al., 2015), respectively. Their gene expression levels were calculated as Fragments per kilobase million (FPKM), using RSEM (v1.2.8) software package (Li and Dewey, 2011).

### Identification of Differentially Expressed Genes and Kyoto Encyclopedia of Genes and Genomes Enrichment Analysis of Differentially Expressed Genes

Based on the negative binomial distribution, DESeq2 was used to identify the differentially expressed genes (DEGs; Love et al., 2014). For this designation of DEGs, the criteria of log_2_ (fold change) are greater than or equal to 1 and *Q*-value (adjusted *p-value)* is less than or equal to 0.05. To assign specific biological pathways to DEGs, the Kyoto Encyclopedia of Genes and Genomes (KEGG) pathway annotation was used based on the KEGG database (Kanehisa et al., 2008). False discovery rates (FDRs) were controlled using established methods (Benjamini and Hochberg, 1995), such that KEGG pathways with FDR < 0.05 were deemed significantly enriched categories.

### Comprehensive Analysis of Transcriptome and Metabolome

In a previous study (Peng et al., 2020), LC-ESI-MS-based untargeted metabolomics was performed on the six samples, and these were taken at 0, 10, 20 DPA for the two cotton lines from the above transcriptome. Here, the differentially accumulated metabolites (DAMs), that is those metabolites that underwent significantly differential accumulation according to these parameters: (1) VIP ≥ 1, (2) fold change ≥1.2 or ≤0.833, (3) *Q*-value < 0.05, were used for comprehensive analysis with the corresponding transcriptome data (refer to Supplementary Tables 1–3). These DAMs’ pathways were then searched against the online database of KEGG pathways.

### Gene Network Construction and Visualization

The weighted gene coexpression network analysis (WGCNA) was performed to identify modules and networks of highly correlated genes based on the FPKM values of all DEGs with the help of an R package (v1.68) (Langfelder and Horvath, 2008). The following calculation parameters were at least 75% of the samples having an FPKM > 1, and soft thresholding power = 7, minimum module size = 30, and minimum height for merging modules = 0.3 were selected to analyze the RNA-seq data sets. After clustering with all samples FPKM, it is found that the two Z161_5-R1 and Z161_10-R2 samples are outlier samples, which can be removed. Finally, a total of 10,891 DEGs were used as input files to participate in the WGCNA. The dynamic tree-cutting algorithm was used to cut the hierarchical clustering tree, and the module was defined after decomposing or merging branches to obtain a stable number of clusters. The model characteristic gene (ME) is defined as the first principal component of a given model. It can be considered as the representative of the gene expression profile for that module. All the modules contained 8,305 genes.

### Identification of the Sample or Stage- and Metabolites Modules

We determined the relationship between each module and cotton phenotypic traits of the samples (sample = 1, all other samples = 0) and metabolites (= 9 flavonoids’ content). Later, the correlations between a module and sample specificity and flavonoids were determined, for which a correlation matrix was drawn in the R software package “ggplot2” (Wickham, 2011). A positive correlation indicated that the genes in a module were expressed preferentially in a specific sample or stage or flavonoids in all other samples.

### Candidate Gene Selection

First, we collected all gene family members (2,285) related to the pathway and TF family members (5,011; refer. http://planttfdb.gao-lab.org/index.php?sp=Ghi) in upland cotton and then counted their numbers as distributed in the different modules. Given that R2R3-MYB, basic helix-loop-helix proteins (bHLH), and WD40 protein (MBW complexes) are related to fiber pigmentation, we focused on the MBW complexes and structural genes. The crucial module networks (kMEmagenta, kMEcyan, and kMElightyellow) containing R2R3-MYB, bHLH TFs, and candidate target genes were visualized using Cytoscape (v3.7.1, United States).

### Identification of Conserved Motifs in Promoter and Yeast One-Hybrid Assay

Conserved motifs of five structural genes in the flavonoid biosynthesis (FB) pathway were investigated using the website toolkit Multiple Expectation maximization for Motif Elicitation (MEME 5.3.3). The optimized parameters of MEME were as follows: site distribution, zero or one occurrence per sequence; number of motifs, 20; and motif width, between 6 and 50 (Bailey et al., 2009). Meanwhile, there were nine conserved motifs, of those five genes were further annotated with TBtools (Chen et al., 2020). Yeast one-hybrid (Y1H) experiments were conducted using the Matchmaker Gold Y1H library Screening System (Clontech, Code 630493). The open reading frame (ORF) of three upland cotton MYB genes was successfully cloned, which was named *GhMYB3 (Gh_A05G235400)*, *GhMYB6 (Gh_D13G138800)*, and *GhMYB46 (Gh_D09G124900)* (named after *Arabidopsis* homologs). Based on a structural analysis of the promoter of leucoanthocyanidin dioxygenase (*GhLODX/GhANS*, *Gh_D08G197600*), anthocyanidin reductase *(GhANR1/2*, Gh_A05G152700/*Gh_D05G168500*), and UDP-glucose: flavonoid 3-O-glucosyltransferase (*GhUFGT1/2*, *Gh_A02G169700/Gh_D03G054700*), 13 DNA motifs were assayed *in vitro*. Two oligonucleotides containing tandem copies of three *cis-*regulatory sequences were inserted into the upstream region of the AbAr reporter gene in the pAbAi vector and then transformed into *Saccharomyces cerevisiae* Y1H Gold strain to produce the bait reporter strain. We tested the bait reporter strains on minimal synthetic defined media (SD) lacking uracil with different concentrations of Aureobasidin A to determine the minimal inhibitory concentration of Aureobasidin A for this bait. The full-length coding sequence of *GhMYB3*, *GhMYB6*, and *GhMYB46* was cloned into pGADT7 AD and then transformed into the bait reporter strains to generate different pairwise combinations. After 3–5 days at 30°C, we assessed and scored growth on the minimum synthesis-limited medium (SD) lacking leucine and supplemented with the lowest inhibitory concentration of Aureobasidin A. If the strains grew normally on both media, there was an interaction between the prey plasmid and the corresponding bait gene. On the contrary, if there was normal growth on SD/-Leu medium, yet no growth on the SD/-Leu/ABA* medium indicates null interaction between them. All the primers are listed (Supplementary Table 4), and the results of each step of the yeast one-hybrid assays are shown in Supplementary Figures 1, 2.

### Quantitative Real-Time PCR Analysis

The expression levels of 20 genes involved in the proanthocyanidins biosynthesis were measured in Z161 and RT at 0,5,10,15, and 20 DPA by conducting a Quantitative Real-Time PCR (qRT-PCR) analysis. The first strand cDNAs for the qRT-PCR were synthesized in a 20-μL solution (containing 1 μg of RNA as the template) using the First-strand cDNA Synthesis SuperMix kit (No. E047-01B; Novoprotein, Shanghai, China) and following the manufacturer’s instructions. Later, the qRT-PCR was performed using Novostar^®^ SYBR qPCR SuperMix Plus (Code No. E096-01A; Novoprotein, Shanghai, China) in a LightCycler 480 (Roche, Mannheim, Germany). The housekeeping gene was *GhUBQ*. The gene-specific primers were designed using the Primer-BLAST online tool^2^, and all the primers are listed in Supplementary Table 5. Each qRT-PCR analysis was performed in three biological replicates, each with three technical replicates. The relative expression values of these genes were calculated according to the 2^–ΔΔCT^ method (Livak and Schmittgen, 2001).

### Data Analysis

Statistically significant differences in significance for the agronomic traits data between the two cotton genotypes were determined by two-way ANOVA, and these results were presented with the help of GraphPad Prism version 9 (GraphPad Software, Inc., San Diego, CA, United States). The online analysis platform OmicShare^3^ was used for the KEGG enrichment analysis. Dot charts were used to depict the number of genes and KEGG enrichment analysis of DEGs, drawn using the R package “ggplot2.” The heatmap for gene expression level was drawn with TBtools (Chen et al., 2020).

### Availability of Data and Materials

The RNA-seq raw data set used in this study has been uploaded to Sequence Read Archive^4^ under BioProject PRJNA766762. The metabolome raw data set was submitted to the Metabolite database^5^ and has the project ID MTBLS2715.

## Results

### Characteristics of the Phenotypic and Transcriptomic Data of Brown and White Cotton Genotypes

**FIGURE 1:**
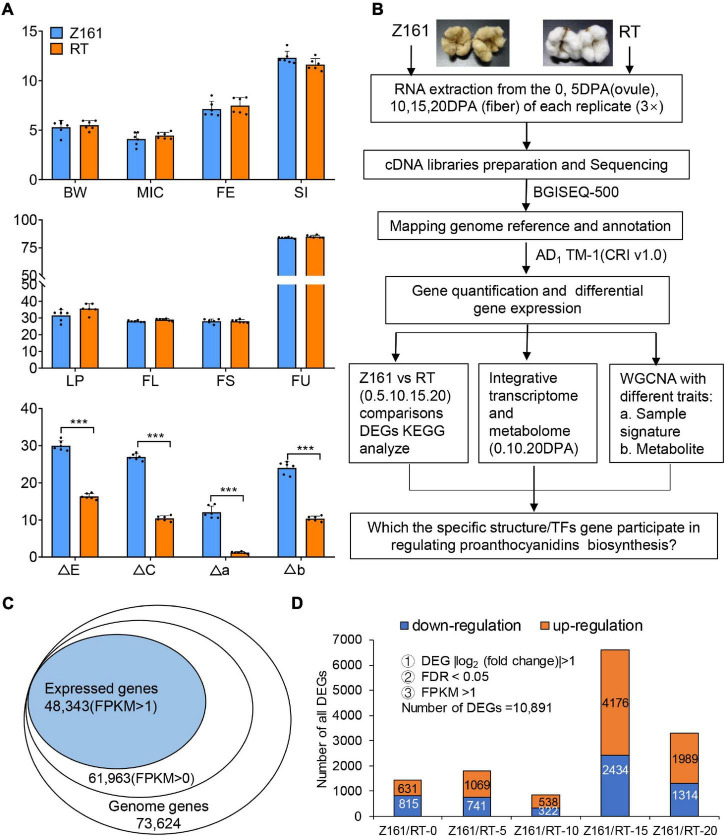
Analysis of agronomic characters and transcriptome data of Z161 and RT at different cotton fiber development stages. **(A)** Analysis of eight agronomic characteristics, boll weight (BW), micronaire (MIC), fiber elongation (FE), seed-index (SI), lint percentage (LP), fiber length (mm) (FL), fiber strength (cN/tex) (FS), fiber length uniformity (FU), and fiber color traits (ΔE, ΔC, Δa, Δb), for 2 years (2018 and 2019) in three environments (Anyang, Alaer, and Kuitun). **(B)** Flow chart of the specific structure/TFs genes identified *via* transcriptomic and metabolic analyses. **(C)** Proportion of expressed genes identified in the *G. hirsutum* reference genome’s gene database. **(D)** Number of DEGs between Z161 and RT at five fiber development stages. The significant difference is Student’s *t* test; *p < 0.05; **p < 0.01; ***p < 0.001.

As noted before (Peng et al., 2020), the two cotton lines used in this study are a pair of NILs. No significant differences were detected in the agronomic traits between the two genotypes at maturity, except for their fiber color (Figure 1A). Specifically, we found significant differences in four indexes of fiber color between the two lines. These findings pave the way to further analyze the pertinent genes functioning to confer brown cotton its fiber pigmentation.

BGI Tech (Shenzhen, China) sequencing technology was used for comparative transcriptome analysis of the Z161 and RT cotton plants at the fiber development stage. Metabolomics is a non-targeted strategy to monitor the multiple changes among different samples. Figure 1B provides an overview of the metadata analysis strategies used in this study. After conducting a quantitative and differential analysis of gene expression levels, we carried out an in-depth analysis of three aspects. Finally, the specific TFs genes involved in the regulation of proanthocyanidins biosynthesis were screened out.

To further characterize the expression levels of pigmentation-related genes in BCF, the two cotton genotypes with contrasting fiber color at five developmental stages (0, 5, 10, 15, and 20 DPA) underwent a transcriptomic analysis. A total of 197.63-Gb high-quality base pairs were obtained, with an average of 6.59-Gb data per sample (Supplementary Table 6). Approximately 92.05–95.25% of the reads were mapped onto the *Gossypium hirsutum* genome, among them, of which 56.71–72.46% were uniquely aligned (Supplementary Table 7). A total of 61,963 genes (FPKM > 0) were detected in the two cotton genotypes sampled at 0–20 DPA. After filtering under the *a priori* specified conditions (FPKM > 1), a total of 48,343 (78.02%) of those genes were identified for further differentially expressed analysis (Figure 1C). These designated 10,891 DEGs related to the pigmentation of the two cotton genotypes were detected. The number of DEGs in either genotype was lowest at the 10 DPA stage and highest at 15 DPA, followed by 20 DPA (Figure 1D and Supplementary Table 8). Hence, by comparing gene expression differences between the two genotypes at the genome-wide level, the results indicated that 15 DPA is the key stage of brown pigment biosynthesis.

### Comparative Transcriptome Analysis of Differential Gene Expression Profiles Between Brown and White Cotton Genotypes

**FIGURE 2:**
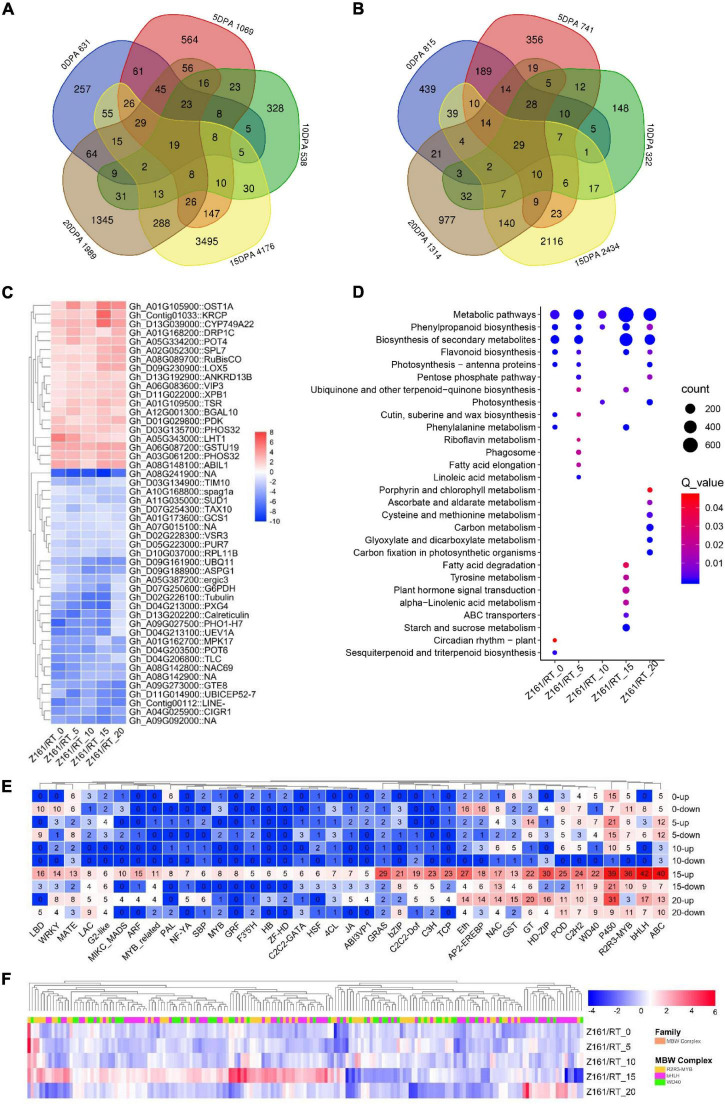
Transcriptomic profiles in Z161 vs. RT and key genes identified in FB. Venn diagram showing the common upregulated **(A)** and downregulated **(B)** DEGs between Z161 and RT among the five stages. **(C)** Heatmap of 19 and 29 DEGs which were continuously upregulated or downregulated between the Z161 and RT during the fiber development stages. **(D)** Analysis of the KEGG enrichment of DEGs in different stages. **(E)** The number of upregulated and downregulated DEGs corresponding to different family members (including TF and structural genes) related to FB. **(F)** Heatmap of 185 MBW complexes of DEGs between Z161 and RT at the five stages. All information for these DEGs can be found in Supplementary Tables 4, 5.

Using the dual criteria of a 95% confidence level (FDR < 0.05) and a minimum 2-fold change, 10,891 (14.79% of 73,624) DEGs were identified in both Z161 and RT genotypes at five developmental stages (Figure 1D). A Venn diagram analysis was used to illustrate the distribution of upregulated and downregulated DEGs and their overlap between the Z161 and RT genotypes (Figures 2A,B). This revealed 19 and 29 common DEGs continuously upregulated and downregulated in the five stages, respectively (Supplementary Table 9). Among them, the upregulated genes were cytochrome P450 (Gh_D13G039000), squamosa promoter-binding-like protein (Gh_A02G052300), WD repeat-containing protein (Gh_A06G083600), glycosyltransferase protein (Gh_A01G105900), glutathione S-transferase (Gh_A06G087200), ABI interactor-like protein (Gh_A08G148100), etc., whereas the downaccumulated genes were cysteine synthase (Gh_A08G241900), probable peroxygenase (Gh_D04G213000), tubulin (Gh_D02G226100) phosphate transporter (Gh_A09G027500), polyubiquitin (Gh_D09G161900), etc (Figure 2C).

To better understand the enriched pathways related to the biosynthesis and metabolism of DEGs, a KEGG pathway enrichment analysis was performed using an FDR < 0.05. In this way, 28 significantly enriched KEGG pathways (Figure 2D andSupplementary Table 10). Evidently, the top-five KEGG pathways were phenylpropanoid biosynthesis, FB, starch and sucrose metabolism, plant hormone signal transduction, and phenylalanine metabolism except for metabolic pathways and secondary metabolite biosynthesis, which suggested their involvement in fiber pigmentation and development at 15 DPA in both genotypes.

Next, we sought to identify and screen the specific members of the structural genes and regulatory genes in the pigment biosynthesis pathway of brown cotton during its fiber development stage. To do this, we first identified all the structural genes and MBW complexes in the proanthocyanidin biosynthesis pathway, and also the genome-wide TF family members. Then, we cross analyzed with the DEGs in the five stages between Z161 vs. RT, and the total of 1,568 DEGs’ information was obtained (Supplementary Table 11). It was found that the top-ten families were bHLH, ABC, P450, R2R3-MYB, HD-ZIP, GRAS, Eth, POD, C2H2, and C3H, especially at the 15 and 20 DPA stages (Figure 2E). Multiple members of the MBW complex were observed. Other research has shown that the R2R3-MYB, bHLH, and WD40 TFs and MBW complexes play an important role in the biosynthesis of anthocyanins and proanthocyanidins (Xu et al., 2015). Compared with the white cotton RT, the MBW family members of brown cotton had more DEGs at 15 DPA, with some members having a greater abundance of DEGs at other stages (Figure 2F). These results confirmed that 15 and 20 DPAs during the process of BCF development were the key periods of active transcription of genes regulated by the pathway of pigment biosynthesis.

### Comprehensive Analysis of the Transcriptome and Metabolome of Cotton

**FIGURE 3:**
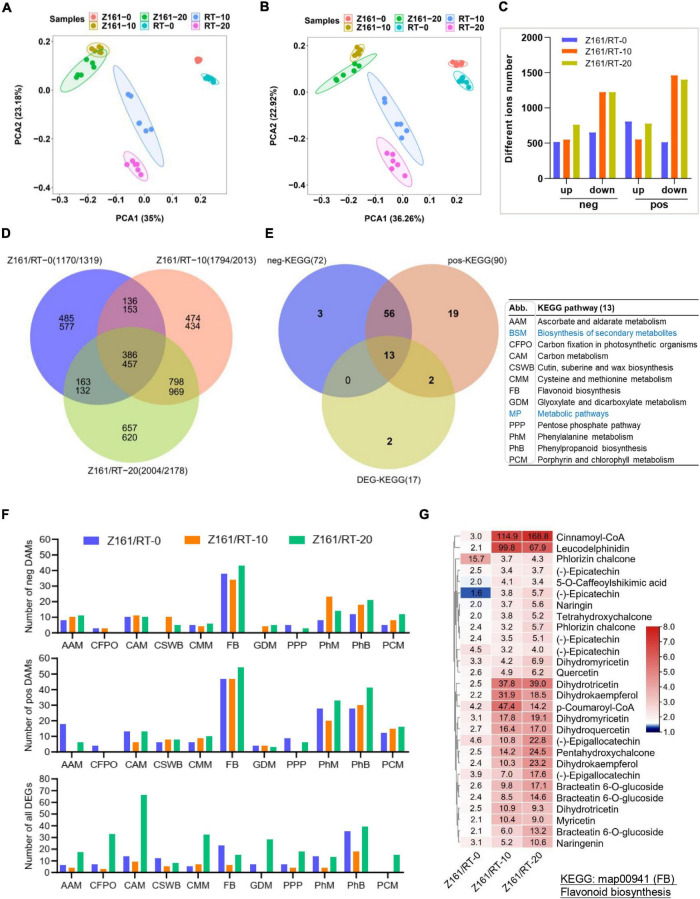
Association analysis between transcriptome and metabolome during fiber development in Z161 vs. RT. PCA of samples (with six biological repetitions) at 0, 10, and 20 DPA, between Z161 and RT in the neg- **(A)** and pos- **(B)** ion mode. **(C)** Numbers of upregulated and downregulated DAMs in negative and positive ion mode between Z161 and RT at the same stage. **(D)** Venn diagram showing the common of upregulated and downregulated DAMs between Z161 and RT. **(E)** Venn diagram showing the unique and common KEGG pathways and all the corresponding pos- and neg-DAMs mapped to the KEGG pathways between Z161 and RT. The 13 pathways abbreviations are explained in the accompanying table. **(F)** Numbers of DAMs and DEGs from these 11 metabolic pathways in the three comparisons. **(G)** Heatmap of 28 representative DAMs in FB. Blue labeled metabolic pathways are the two largest classifications.

The untargeted metabolomic data were first used for principal component analysis (PCA). Six groups were clearly separated on the PC1 × PC2 score plot with neg- and pos-model (Figures 3A,B). Our results showed that the raw data from the comparisons of the two cotton lines’ comparison at the same stage could reflect the significant differences between the samples. The PLS-DA method (Wen et al., 2017) was used for multivariate analysis of the six comparisons, to identify those DAMs with statistical and biological significance and thereby elucidate the related metabolic processes. The number of DAMs showed a similar trend in both two ion modes. At 10 and 20 DPA (fiber elongation phase), the accumulation level of metabolites decreased more than that at 0 DPA (fiber initiation phase). At the same time, the accumulation level of metabolites increased more among different genotypes at 20 DPA (Figure 3C). The results also uncovered the highest number of specific upregulated and downregulated metabolites in the overlapping phases of fiber elongation and secondary wall thickening (20 DPA), suggesting that this was the most active period for the deposition of various metabolites of brown cotton and white cotton (Figure 3D). To further investigate the DAMs with DEG-enriched pathways, a cross analysis of the DEG-enriched KEGG pathways (17) and the corresponding pathways of neg (90) and pos- (72) DAMs were mapped, with common 13 metabolic pathways (Figure 3E and Supplementary Table 12). As shown in Figure 3F, the top-five KEGG pathways were phenylpropanoid metabolism, FB, phenylalanine metabolism, carbon metabolism, and cysteine and methionine metabolism, which suggested that these DEGs in both genotypes were mainly enriched in five pathways compared with different development stages at the metabolomic and transcriptional levels. The heatmap for the differences in flavonoid content among the three comparisons indicated the greater levels of flavanols in the fiber of Z161 than in RT at 10 and 20 DPA (Figure 3G and Supplementary Figure 3). To sum up, these results indicated that gene expression and related metabolite production in flavonoid pathways were more active from the early stage of fiber initiation development stage.

### Identification of Key Genes and Regulators Related to Proanthocyanidins Biosynthesis in Brown Cotton

**FIGURE 4:**
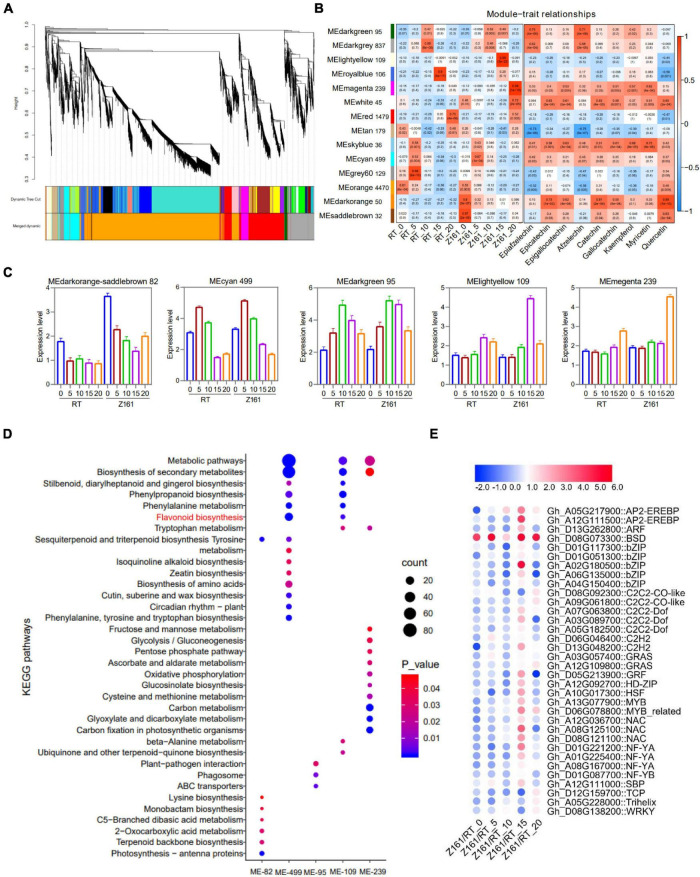
Weighted gene coexpression network analysis (WGCNA) for DEGs. **(A)** Hierarchical cluster dendrogram. **(B)** Module–trait relationships with five stage samples of two varieties (Z161 and RT) and flavonoid contents. **(C)** Eigengene expression profile for the six modules in five-stage samples of two varieties (Z161 and RT). **(D)** KEGG enrichment for the six modules genes. **(E)** Expression pattern analysis of TF genes from the six modules. All information for these genes can be found in Supplementary Tables 11, 12. The metabolic pathway marked by red is a metabolic pathway related to pigment formation.

To investigate the gene coexpression and regulatory network of proanthocyanidins biosynthesis in brown cotton, WGCNA was conducted using 10,891 filtered DEGs (Supplementary Table 8). Overall, 14 distinct coexpression modules corresponding to clusters of correlated genes were detected (Figure 4A and Supplementary Table 13). Each stage of the cotton samples and the fold-change of different flavonoids content of the three comparisons of metabolome behavior were used as trait data for a module–trait relationship analysis (Figure 4B). The MElightyellow module (109 genes) presented the strongest correlation with Z161-15 (*r* = 0.99, *p* = 2e-22) whereas the MEmagenta modules (239) were most corrected with Z161-20 (*r* = 0.98, *p* = 1e-19). Also, selected were MEdarkorange and MEsaddlebrown (82 genes), and MEcyan (499) and MEdarkgreen (95), based on the different stage samples of Z161 and the different differing abundance of flavonoids with *r-values* > 0.5. A summary profile for each module was based on the average value of FPKM from each sample, and that highly correlated traits are shown in Figure 4C. Furthermore, the KEGG pathway enrichment analysis of the above six modules showed that the FB and phenylpropanoid metabolism were significantly enriched in the MEcyan and MElightyellow module (Figure 4D). Except for the MBW complex, 34 TF genes of 20 families were screened, most of which were expressed more in 15 DPA than in other stages (Figure 4E and Supplementary Table 14). Among them, *GhBSD* (Gh_D08G073300) gene was highly expressed in all five stages of Z161 (0–20 DPA).

**FIGURE 5:**
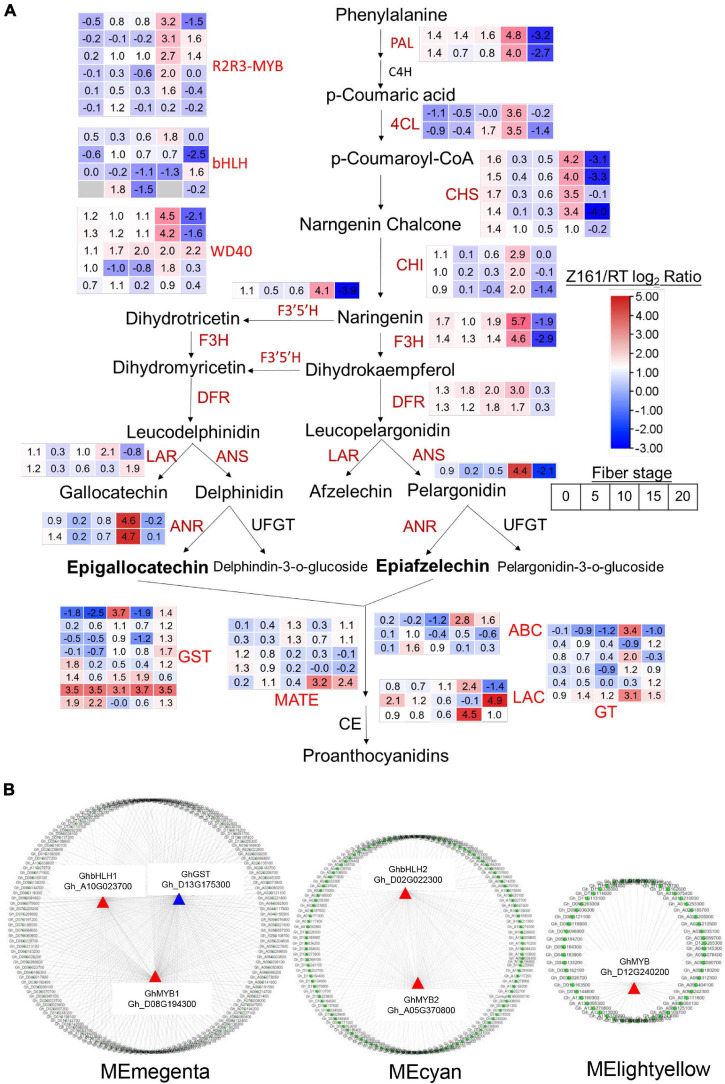
Analysis of the structural and TF DEGs for FB in Z161 vs. RT. The pathways were constructed based on the KEGG pathways and literature sources. **(A)** Red fonts indicate upregulated genes (catalytic enzymes and TF) in Z161 during at least one stage of fiber development. Gene expression patterns are displayed in the box with a red–blue color model. FB (map00941). PAL, phenylalanine ammonia-lyase; C4H, cinnamate 4-hydroxylase; 4CL, 4-coumarate-CoA ligase; CHI, chalcone isomerase; CHS, chalcone synthase; F3′5′H, flavonoid 3′,5′-hydroxylase; F3H, flavanone 3-hydroxylase; F3′H, flavonoid 3′-monooxygenase; DFR, dihydroflavonol-4-reductase; LAR, leucoanthocyanidin reductase; ANS, anthocyanidin synthase; ANR, anthocyanidin reductase; UFGT, UDP flavonoid glucosyl transferase; GT, anthocyanidin 3-O-glucosyltransferase; GST, glutathione S-transferase, MATE, Multidrug and toxic compound extrusion or multiantimicrobial extrusion; ABC, ATP-binding cassette; LAC, laccase; GT, glycosyltransferase; CE, condensation enzyme. **(B)** Cystoscope representation of coexpressed genes with edge weight ≥0.20 in “magenta,” “cyan,” “lightyellow” modules. Coloring shows the node R2R3-MYB or bHLH genes. All the DEGs are shown in Supplementary Table 14.

**FIGURE 6:**
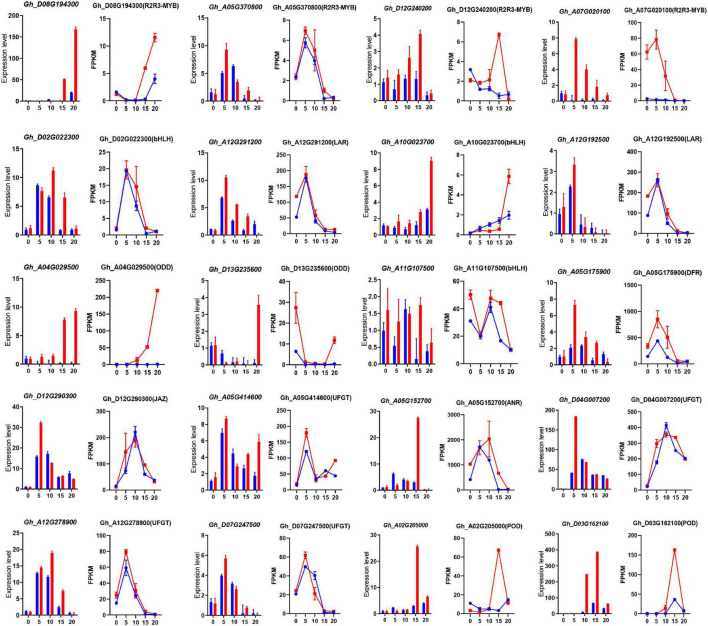
The qRT-PCR analysis of the expression of 20 pigmentation-related candidate genes between Z161 and RT at the five stages. Relative expression levels are presented as mean ± SD, *n* = 3. The corresponding FPKM values were derived from the RNA-seq data. All the primers are shown in Supplementary Table 5. Red and blue pillars or lines represent Z161 and RT, respectively.

To understand the differential expression of structural genes and regulatory genes in the FB pathway, 290 genes from 15 catalytic enzyme or transporter families and MYB-bHLH-WD40 (total 62 genes) from the above five ME modules were identified (Figure 5A and Supplementary Table 15). It is worth mentioning that the two *GhANS* (Gh_A08G1593) and *GhANR* (Gh_A05G1424) genes were also highly expressed in the Z161 proteome data (see Figure 5A from Peng et al., 2020). In tandem, we also identified some important glutathione-S-transferase (GST), multidrug and toxic compound extrusion (MATE), laccase (LAC), ABC transporter (ABC), and glucosyltransferase (GT) genes and they play a key role in the process of transporting proanthocyanidins to vacuoles for further condensation and polymerization. According to the above five modules, we tried searching for the coexpression networks of the R2R3-MYB and bHLH hub genes, thereby finding three important networks (Figure 5B and Supplementary Table 16). In MEmagenta, *GhMYB1* (Gh_D08G194300), *GhbHLH1* (Gh_A10G023700), and *GhGST* (Gh_D13G175300) were the most correlated genes. In MEcyan, the *GhMYB2* (Gh_A05G370800) and *GhbHLH2* (Gh_D02G022300) genes were the most correlated genes. However, there was only one R2R3-MYB gene *GhMYB* (Gh_D12G240200) that correlated most with other genes in MElightyellow. To verify the reliability of our cotton RNA-seq data, we examined 20 genes possibly involved in the biosynthesis of phenylpropanoid and proanthocyanidins, and their results were also consistent with those of their qRT-PCR analysis (Figure 6).

To conclude, taken together, these results confirmed that the expression levels of some key regulatory genes and structural genes of the flavonoid pathway are far higher in Z161 than RT at 15 and 20 DPA, leading to rapid pigment biosynthesis and polymerization at the end stage of the fiber elongation or secondary wall thickening stage. The coexpression patterns of R2R3-MYB and bHLH with other genes at distinct stages may also play a key regulatory role in cotton fiber pigmentation.

### Three R2R3-MYB Proteins Bind to the Promoters of *GhANS, GhANR1*, and *GhUFGT2* Genes

**FIGURE 7:**
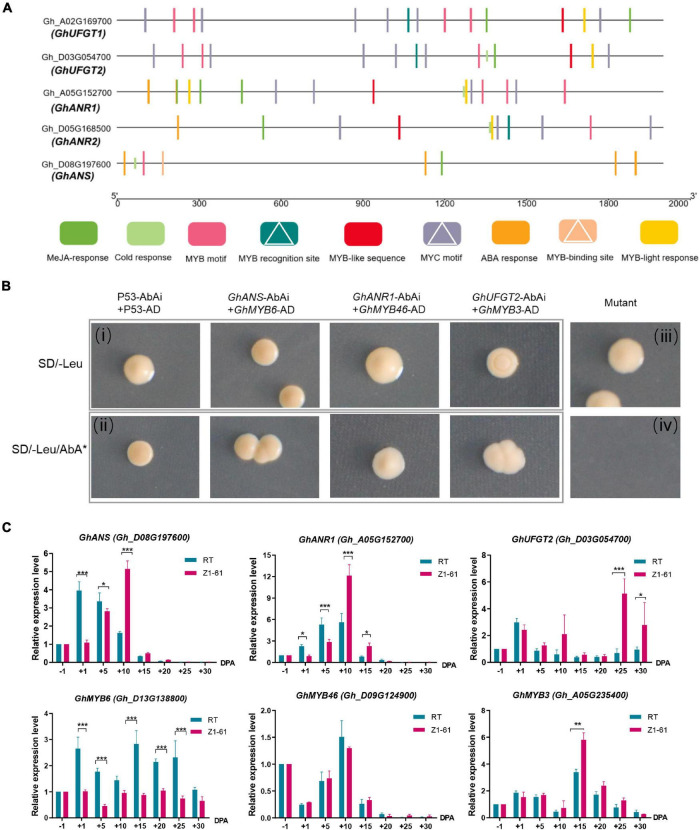
Validation of the interaction of the transcriptional regulatory complex by yeast one-hybrid (Y1H) assays and qRT-PCR. **(A)** Schematic representation of MYB motifs in the promoter regions of the flavonoids biosynthetic genes (*GhANR1*, *GhANR2*, *GhUFGT1*, *GhUFGT2, and GhANS*) from cotton. MYB recognition sites, MYB-binding sites, and MYB motifs are indicated by white triangles. **(B)** Y1H analysis of *GhMYB6*, *GhMYB46*, *GhMYB3*, interactions with the canonical MYB-binding motif, respectively, identified in *GhANS*, *GhANR1*, and *GhUFGT2* promoter sequences, respectively. The **(i,ii)** are combined as a positive control, whereas **(iii,iv)** are combined as a negative control. SD, minimal media; Leu, leucine; AbA, Aureobasidin A. **(C)** qRT-PCR analysis of transcriptional levels of *GhMYB6*, *GhMYB46*, *GhMYB3, GhANS*, *GhANR1*, and *GhUFGT2* genes between Z161 and RT at these fiber development stages (–1, 1, 5, 10, 15, 20, 25, and 30 DPA). All the primers are shown in Supplementary Table 4. The significant difference is Student’s *t* test; *p < 0.05; **p < 0.01; ***p < 0.001.

In our previous study, proteomics distinguished the key proteins *GhANR1* (Gh_A05G1424) and *GhANS* (Gh_A08G1593) as being highly abundant at 10 and 20 DPA in brown cotton Z161. Meanwhile, the qRT-PCR showed that *GhUFG11T2* was expressed from −1 to 30 DPA, and the expression level of 25 and 30 DPA in Z161 was higher than that in other stages (Peng et al., 2020). Likewise, in this study, the expression of three genes – *GhANR1* (Gh_A05G152700), *GhANR2* (Gh_D05G168500), and *GhANS* (Gh_D08G197600) – underwent more than a 16-fold change in Z161 vs. RT at transcriptional level at 15 DPA. Interestingly, both the identified genes (*GhANR1* and *GhANS*) exhibited the same expression trend as inferred from the proteome analysis (Figure 5A). To verify whether the five key genes of proanthocyanidin biosynthesis may be transcriptionally regulated by R2R3-MYB TF, the possible MYB-binding motifs in the promoter region of these genes were first predicted (i.e., the 2K sequence upstream of the gene transcript initiation sites). Later, the MYB recognition site [CNGTT(A/G)], MYB motif [T(C)AACCA], MYB-binding site (CAACAG), and MYB-like sequence (TAACCA) were identified in the promoter region (Figure 7A). The binding ability to the promoters of key structural genes *GhANR1/2*, *GhANS*, and *GhUFGT1/2* was then investigated using the yeast one-hybrid (Y1H) assay.

The ORF of GhMYB6 was inserted into MCS of pGADT7 vector to serve as the prey construct, which was cotransformed with the individual bait constructs harboring different MYB-binding motifs (these cloned from *GhANR1/2*, *GhANS*, and *GhUFGT1/2* promoter sequences) fusion proteins. This demonstrated that GhMYB6 could only physically interact with *GhANS* (Figure 7B), but no physical interactions occurred between GhMYB6 and *GhANR1*, *GhANR2*, or *GhUFGT1/2* (data not shown). In checking for possible interactions *via* different comparisons, it was finally determined that *GhMYB3* and *GhMYB46* can only physically interact with *GhUFGT2* and *GhANR1*, respectively (Figure 7B). Expression trends of the *GhANR1*, *GhANS*, and *GhUFGT2* genes and their corresponding regulatory genes —*GhMYB46*, *GhMYB6*, and *GhMYB3* —during fiber development were analyzed by qRT-PCR (Figure 7C). Three R2R3-MYB proteins could bind to the promoters of *GhANS*, *GhANR1*, and *GhUFGT2*. It is worth noting that GhMYB6 might negatively regulate the transcription of the *GhANS* gene, but this needs to be verified by further research.

## Discussion

The color of NCC results from the accumulation of pigments during the differentiation and development of fiber cells. Previous research has mainly focused on the fiber pigmentation (at ∼20 DPA; fiber elongation and secondary wall thickening). In this study, the data for the transcription and metabolism of white and brown fibers were analyzed in the early and late elongation stage and during the stage of secondary wall thickening. These studies are helpful for exploring the regulatory network mechanism of TFs of the proanthocyanidins biosynthesis pathway in brown cotton plants.

### Key Period of Transcription Regulation of Pigmentation-Related Genes in Brown Cotton Fiber Development Occurs From 15 to 20 Days Post-anthesis

As is well known, both white cotton and colored cotton go through four stages of fiber development: differentiation and protrusion of fiber primordial cells, elongation of primary wall, thickening of the secondary wall, and dehydration and maturation. But colored cotton undergoes an additional process of pigment synthesis and deposition (Murthy, 2001). Many recent studies have shown that PAs are the main component of pigment deposition in BCF (Li et al., 2013; Feng et al., 2014; Xiao et al., 2014; Yan et al., 2018), and their content reaches typically peaks at 30 DPA (Feng et al., 2014). The biosynthesis of proanthocyanidins precursors regulates the FB pathway to mediate the gene expression of flavonoid metabolism pathway for the development of BCF (Hua et al., 2007; Xiao et al., 2007, 2014; Feng et al., 2013; Li et al., 2013; Tan et al., 2013; Gao et al., 2019). Earlier research demonstrated that the synthesis of proanthocyanidins in fiber is the main reason for the shortened elongation period, thus halting the early elongation of BCF (Qiu, 2004). The dominant expression of genes related to the biosynthesis of proanthocyanidins precursors will play a vital role in the fiber development stage. In this study, we compared the transcriptome of brown cotton and its white cotton NILs at 0, 5, 10, 15, and 20 DPAs. The results showed that 15 and 20 DPAs corresponded to the peak period for the dominant expression of genes related to the FB pathway during fiber development of brown cotton. These above said findings were substantiated by the analysis of differentially expressed structural genes and TFs (triple complex) related to FB and by the weighted gene coexpression network. Xiao et al. (2007) were able to detect key genes in cotton by semiquantitative PCR, finding their greatest expression at 16 DPA and decreased at 20 DPA. Likewise, Canavar and Rausher (2021) recently reported that structural gene expression reached its peak at 14 DPA. Additionally, Peng et al. (2020) revealed that 20 DPA is the peak time for the accumulation of important proteins and metabolites in the biosynthesis pathway of flavonoids. Therefore, the proteins and metabolites accumulated at 20 DPA may be the genes highly expressed at 15 DPA in the posttranscription, translation, and posttranslation stages.

### Gene Regulatory Network Related to Flavonoid Biosynthesis Pathway During Brown Cotton Fiber Development

Few reports exist that comparatively analyze main flavonoids metabolites in the fibrous tissues of brown cotton and white cotton at different developmental stages. In this study, all metabolites in the flavonoid pathway were identified and quantitatively expressed by a non-targeted metabolome analysis. Comparative analysis revealed that the main metabolites (i.e., flavanediol and flavane-3-alcohol) accumulated substantially in BCF. Previous studies have shown that the final pigment component in BCF is mainly PA. Flavane-3-ol is the precursor unit (flavonoids) of proanthocyanidins polymerization (Feng et al., 2014), and we further confirmed that a large number of pigment precursors (flavonoids) were accumulated during the BCF elongation stage of brown cotton (10–20 DPA). Previously, Feng et al. (2013) had shown that the accumulation of naringenin, quercetin, kaempferol, and myricetin in brown fiber at 12–21 DPA significantly exceeded that in white cotton fiber by HTLC analysis. That finding is consistent with our metabolome results.

Further, we explored differences in gene expression in five developmental stages *via* paired comparison of transcriptome data. The results of the DEG analysis in different periods demonstrated that some specifically expressed genes involved in flavonoid synthesis pathway were enriched in the four periods at 0, 5, 15, and 20 DPA. It should be noted that the number of upregulated genes of different gene family members involved in the flavonoid synthesis pathway during fiber elongation (5–20 DPA) surpassed that of downregulated genes. In particular, the expression levels of structural genes involved in the flavonoid pathway (e.g., *GhF3H*, *GhLAR*, and *GhANR*) were significantly higher in the BCF samples. These patterns of gene expression patterns are consistent with the findings of Li et al. (2020) and Canavar and Rausher (2021).

Although there are many studies of flavonoid synthesis and its functional genes, the complex regulatory mechanism is still a mystery. Here, the WGCNA method was used to detect the gene interaction network involved in FB. We screened five R2R3-MYB and bHLH hub genes to better understand the *in vivo* relationships between genes during the development of BCF and flavonoid synthesis in the two cotton varieties. In this respect, *GhMYB1* (Gh_D08G194300) and *GhMYB3* (Gh_D12G240200) are homologous to *AtMYB42*, which can directly activate transcription inhibitors, thereby specifically inhibiting FB and directly activating lignin biosynthesis genes (Geng et al., 2020). However, in our study, the gene was upregulated at 15 and 20 DPA in brown cotton (Z161) and is thus speculated to harbor different functions. Building on our previous work (Peng et al., 2020), we also verified that the potential interacting TFs of GhANR1 (homologous gene *GhANR2*), *GhANS*, and *GhUFGT2* (homologous gene *GhUFGT1*) were *GhMYB46*, *GhMYB6*, and *GhMYB3*, respectively. According to their expression patterns, we may infer that *GhMYB6* is a negative regulator and *GhMYB3* is a positive regulator. We anticipate this study’s compelling evidence, and findings can be used to reveal the complex transcriptional regulation mechanism of flavonoid synthesis in BCF in future work.

## Conclusion

In this study, 10,891 DEGs related to the pigmentation of the brown cotton genotypes were detected. It was found that the top-three KEGG pathways at 15 DPA were phenylpropanoid biosynthesis, FB, and starch and sucrose metabolism. According to the DAMs and DEGs distinguishable in different fiber development stages, 13 common key KEGG pathways were identified, which included phenylpropanoid biosynthesis and FB. Compared with RT, the flavonoids contents in Z161 at different stages varied greatly, such as for leucodelphinidin, leucocyanidin, kaempferol, epiafzelechin, epicatechin, and epigallocatechin. Based on the WGCNA, three modules with five TF hub genes were found that were highly correlated with the FB pathway. Furthermore, the three genes *GhANS*, *GhANR1*, and *GhUFGT2* are preliminarily proven to interact with three R2R3-MYB TFs (*GhMYB46*, *GhMYB6*, and *GhMYB3*). The candidate genes and interaction patterns of PAs accumulation in brown cotton elucidated here can provide valuable clues for improving brown cotton’s molecular breeding in the near future.

## Data Availability Statement

The original contributions presented in the study are publicly available. This data can be found here: https://www.ncbi.nlm.nih.gov/bioproject/PRJNA766762.

## Author Contributions

ZW analyzed and summed all the data and wrote the manuscript. XZ designed the yeast one-hybrid experiment and finished the operation. SH directed the methodology and software analysis. XG, QG, and LW managed and collected the plant tissues. YJ, HL, and ZhaP collected the data and evidence and project administration. AR revised the language. ZheP and XD conceptualized the research program and revised the manuscript. All authors read and approved the final manuscript.

## Conflict of Interest

The authors declare that the research was conducted in the absence of any commercial or financial relationships that could be construed as a potential conflict of interest.

## Publisher’s Note

All claims expressed in this article are solely those of the authors and do not necessarily represent those of their affiliated organizations, or those of the publisher, the editors and the reviewers. Any product that may be evaluated in this article, or claim that may be made by its manufacturer, is not guaranteed or endorsed by the publisher.

## Footnotes

1
https://github.com/BGI-fexlab/SOAPnuke

2
http://www.ncbi.nlm.nih.gov/tools/primer-blast/

3
http://www.omicshare.com/tools

4
https://www.ncbi.nlm.nih.gov/sra

5
https://www.ebi.ac.uk/metabolights/

## Supplementary Material

Supplementary Table 1 The different abundance metabolites (DAMs) among negative and positive ions at 0 DPA.

Supplementary Table 2 The DAMs among negative and positive ions at 10 DPA.

Supplementary Table 3 The DAMs among negative and positive ions at 20 DPA.

Supplementary Table 4 Primer sequences used in yeast one-hybrid analysis.

Supplementary Table 5 Primer sequences used in qPCR analysis.

Supplementary Table 6 RNA-seq data statistic.

Supplementary Table 7 Mapping results of all 30 RNA-seq libraries to the *Gossypium hirsutum* reference genome.

Supplementary Table 8 The detailed information of DEGs.

Supplementary Table 9 The detailed information of 19 and 29 DEGs which were continuously upregulated or downregulated between the Z161 and RT.

Supplementary Table 10 DEGs enriched in KEGG pathways.

Supplementary Table 11 The detailed information of 1,568 DEGs (including TF and structure genes) related to FB.

Supplementary Table 12 All DAMs and DEGs enriched in KEGG pathways.

Supplementary Table 13 The detailed information of 8,305 genes of each sample/metabolite-specific module.

Supplementary Table 14 The detailed information of TF genes (exclude MBW genes) of each sample/metabolite-specific module.

Supplementary Table 15 The detailed information of 290 structural and TF genes related to FB of each sample/metabolite-specific module.

Supplementary Table 16 The hub genes of “magenta,” “cyan,” “lightyellow” module.
