# Supplementary material for: Transcriptome Co-expression Network and Metabolome Analysis Identifies Key Genes and Regulators of Proanthocyanidins Biosynthesis in Brown Cotton
Source: Front Plant Sci. 2022 Feb 14;12:822198. doi: 10.3389/fpls.2021.822198 (PMC8882990; doi:10.3389/fpls.2021.822198)
Supplement: Supplementary file 2 [file Data_Sheet_1.PDF]

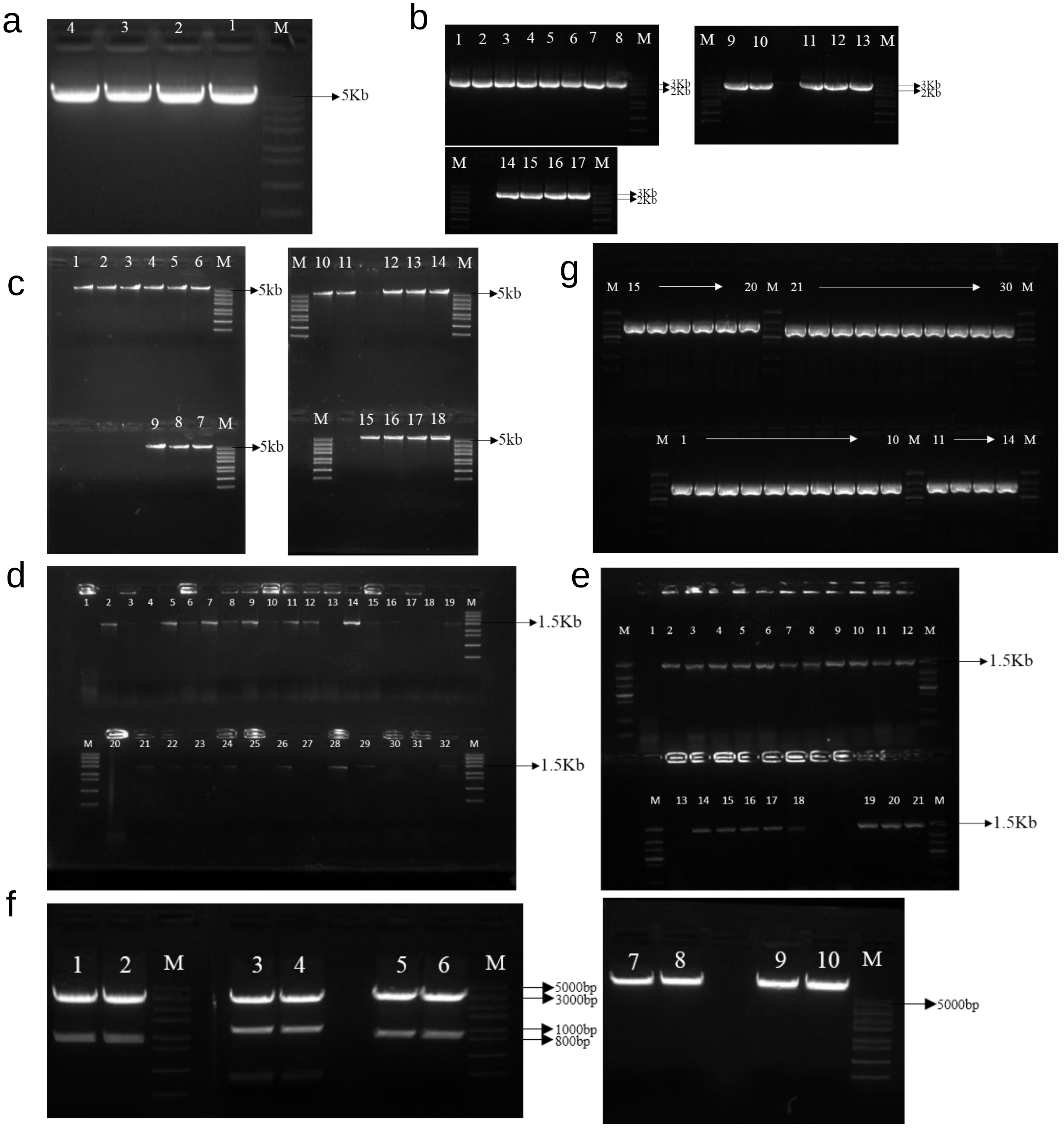

Figure S1: Yeast one-hybrid. (a) Restriction enzyme digestion of pAbAi vector (4895bp) M: *Trans* 5K DNA Marker;1-4: digested pAbAi vector.(b) The right Sequenced Bait-AbAi (including mutation) plasmids. M:Trans 5K DNA Marker; 1 and 6 are Mutant (GhANR1)/Abai plasmids, and 2-5 are GhANR1/Abai plasmids; 7 is mutant (GhDFR)/Abai plasmid, 8 is GhDFR/Abai plasmid; 9-13 is GhANS/Abai plasmid; 14-16 are GhUFGT2/Abai plasmids and 17 are mutant (GhANR1) / Abai plasmids. (c). Single endonuclease digestion of BstBI. 1-18:Bait/AbAi (including mutation) and p53/AbAi plasmid. (d) Yeast bacterium solution PCR product test. 1 and 20 were negative control Y1HGold bacterial solution.16-19 and 29-32 were positive controls p53/Abai. The rest are transformed target gene Bait/Abai (including mutation).(e) yeast bacterium solution PCR product test. 1 and 13 were negative control Y1HGold bacterial solution.8-12 and 19-21 were positive controls p53/Abai. The rest are transformed target gene Bait/Abai (including mutation).(f). Restriction enzyme digestion of target genes and pGADT7-AD vector (GhMYB6. GhMYB3, GhMYB46). 7,8 are NdeI and BamHI double enzyme digestion ; 9 and 10 are EcoRI and BamHI double enzyme digestion of pGADT7-AD vector.(g) Bacterium solution PCR result after linked with pGADT7-AD vector.

Structural gene	NO.	AbA(ng/ml)
Positive control	P53	100
GhDFR	1	100
GhDFR-mutant	2	100
GhANS-1	3	100
GhANS-1-mutant	4	100
GhANS-2	5	100
GhANS-3	6	400
GhANS-4	7	Automatic activation
GhANS-5	8	300
GhANR1-1	9	300
GhANR1-2	10	200
GhANR1-2-mutant	11	Automatic activation
GhANR1-3	12	100
GhANR1-4	13	400
GhANR1-4-mutant	14	Automatic activation
GhUFGT2-1	15	200
GhUFGT2-2	16	200
GhUFGT2-3	17	100
GhUFGT2-3-mutant	18	100

Figure S2: The Results for AbA^r basal expression

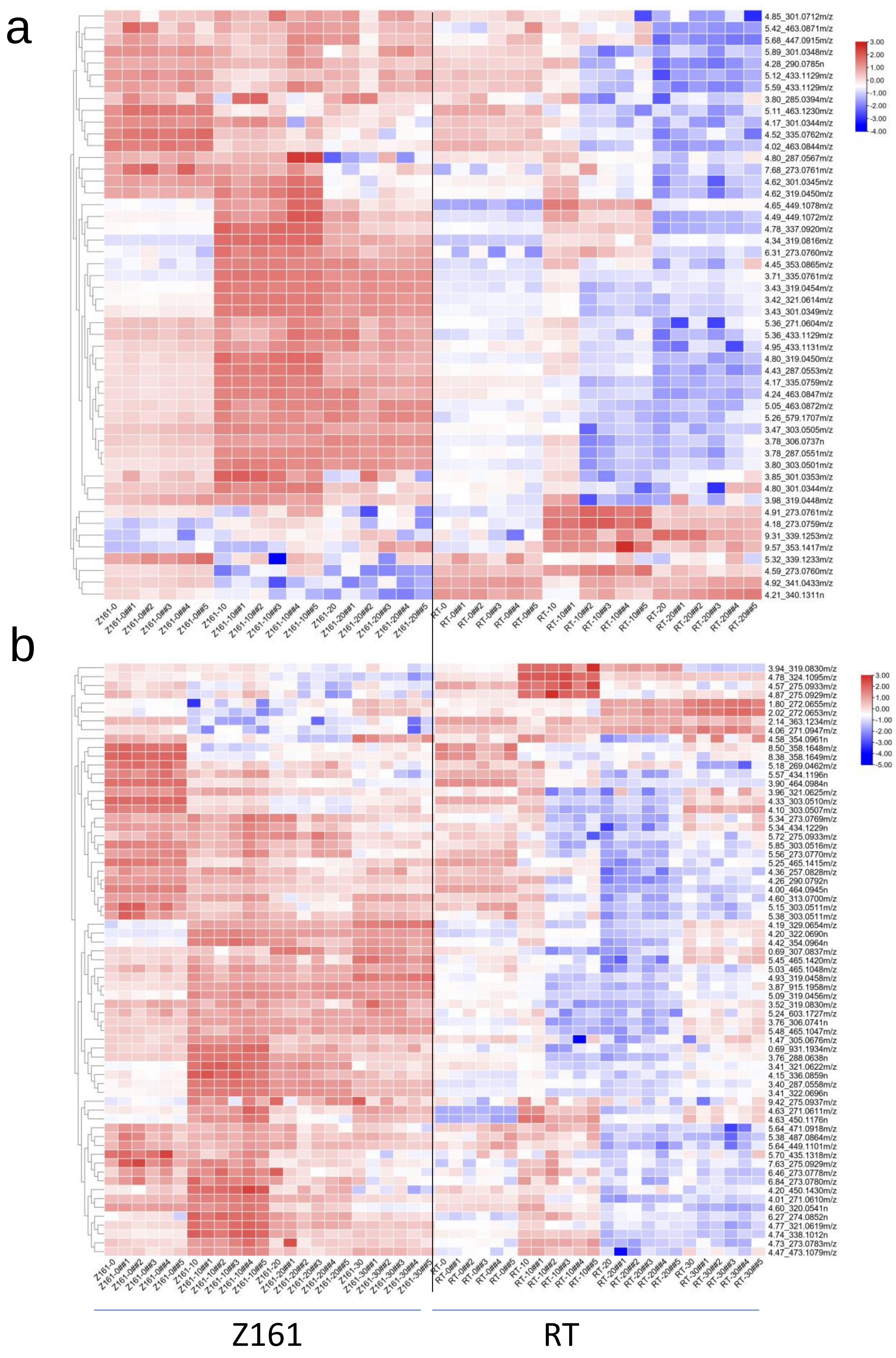

Figure S3: Comparison of PA and flavonoid concentrations between Z161 and RT in fibers at different developmental stage(0.10.20DPA). The data are normalized by Z-score. These metabolites These metabolites come from flavonoid metabolic pathway(map00941).(a) neg-mode. (b) pos-model
